# Susceptibility to digital health misinformation: a multi-level narrative review

**DOI:** 10.3389/fpubh.2026.1801278

**Published:** 2026-04-29

**Authors:** Amna Alabri

**Affiliations:** Department of Mass Communication, College of Creative Industries, University of Technology and Applied Sciences, Nizwa, Oman

**Keywords:** cognitive determinants, digital health misinformation, digital platforms, health literacy, relational and social capital, susceptibility, worldview and identity

## Abstract

Digital platforms have transformed access to health information while also enabling the rapid spread of health misinformation. This narrative review synthesizes evidence from individual, relational, and platform perspectives to provide a multi-level analysis of susceptibility to digital health misinformation. It examines how susceptibility is conceptualized and measured, then organizes determinants across the three domains. At the individual level, susceptibility is associated with limited health, digital, eHealth, and news literacy; reduced cognitive reflection and numeracy; heightened emotional responses; and identity-driven reasoning. Relational factors—particularly trust in clinicians and scientific institutions—are protective, whereas reliance on peer networks, social media, or alternative health communities increases risk. At the platform level, algorithmic curation, passive news exposure, information overload, and AI-generated content shape both susceptibility and sharing behavior. Cross-level interactions further modulate vulnerability. The review concludes with targeted implications for intervention and future research.

## 1 Introduction

The global proliferation of digital health information presents a defining paradox. The same technologies that have democratized access to medical knowledge have become powerful conduits for health misinformation on an unprecedented scale. Across social media platforms, messaging applications, AI-generated content, and algorithmically curated feeds, billions of people now encounter health information through channels designed to maximize engagement rather than ensure accuracy. These channels are largely unregulated and emotionally charged (1, 2). The consequences extend far beyond individual misbeliefs. Health misinformation undermines vaccination campaigns, delays care-seeking, erodes institutional trust, and contributes to avoidable morbidity and mortality (3, 4). These harms fall unevenly across patients, caregivers, providers, and public health systems. These dynamics became most acutely visible during the COVID-19 pandemic, when misinformation shaped risk perception, institutional trust, and health behavior at a population scale (5–9).

Vulnerability varies as a function of cognitive abilities, literacy, emotional processing, motivation, and trust. It is further shaped by the digital platform environment, which influences how individuals process, accept, reject, or disseminate misleading content (2, 10–15). Previous reviews have examined health misinformation topics, sources, and prevalence—particularly during COVID-19 (5, 16)—but rarely integrate determinants of susceptibility across individual, ideological, relational, institutional, and media levels. This gap impedes the development of effective, theory-driven interventions by leaving unresolved how determinants interact and under what conditions susceptibility translates into belief and behavioral change.

This review addresses that gap by synthesizing empirical evidence on the determinants of susceptibility to digital health misinformation across individual, relational, and platform-level domains, using an explanatory approach that examines how and under what conditions each determinant shapes vulnerability. These insights are directly relevant to a broad range of stakeholders. For clinicians and healthcare providers, it highlights the relational and communicative factors that shape patients' acceptance or rejection of evidence-based information. For public health practitioners and policymakers, it identifies leverage points for intervention design and communication strategy. For platform designers and technology companies, it foregrounds how architectural choices amplify or attenuate susceptibility. For patients, caregivers, and communities, particularly those from historically marginalized or underserved groups, it maps the structural, cognitive, and social factors that contribute to disproportionate vulnerability. For researchers, it clarifies where the evidence base remains incomplete and where future investigation is most needed. Accordingly, this review aims to:

1. Clarify how susceptibility to health misinformation is conceptualized and operationalized in the literature.

2. Identify the key individual-level determinants of susceptibility to digital health misinformation.

3. Examine how relational and social-capital factors function as protective or risk-enhancing influences.

4. Assess platform, environmental, and message-level drivers of susceptibility.

5. Analyze cross-level interaction effects that amplify or attenuate vulnerability.

6. Derive insights to inform targeted intervention design and future research.

## 2 Scope and approach

This review is an integrative, explanatory narrative synthesis—not a systematic review—of determinants of susceptibility to digital health misinformation among the public, patients, and caregivers. The goal is to interpret a heterogeneous evidence base thematically, not to catalog studies or produce pooled effect estimates. Given its narrative structure, this review was not pre-registered. Readers seeking formal systematic treatments of susceptibility determinants are directed to Nan et al. (17); for quantitative estimates of the exposure-to-misbelief effect, to Li and Yang (18); and for a stage-based framework organizing engagement with online misinformation, to Geers et al. (19). Relevant studies for the current synthesis were identified through searches of PubMed/MEDLINE, PsycINFO, Web of Science, Scopus, and Communication Source, using terms spanning health misinformation and false claims, susceptibility and discernment, and digital and social media environments. The synthesis draws primarily on health-specific empirical research, supplemented by foundational work from the broader misinformation literature in psychology and communication science [e.g., (20)], where it provides evidence on cognitive or social mechanisms applicable to health contexts. Evidence is organized by level of analysis: individual, relational, and platform-level.

## 3 Review of the evidence

### 3.1 Conceptualization and operationalization of susceptibility to health misinformation

#### 3.1.1 Defining health misinformation and susceptibility

Health misinformation is commonly defined as health-related information or claims that are false, inaccurate, or misleading when evaluated against the best available scientific evidence. This definition applies whether shared intentionally or unintentionally, although the element of intent is often used to differentiate misinformation from disinformation (1, 2). Definitions of susceptibility vary across disciplines but generally capture an individual's tendency or vulnerability to believe or be influenced by false information (21). Because health topics are high-stakes, emotionally charged, and technically complex, research distinguishes mere exposure from susceptibility. Susceptibility reflects the extent to which individuals (a) judge misinforming claims as accurate or credible, (b) allow these claims to shape their attitudes, intentions, or behaviors, and/or (c) disseminate them to others (14, 17, 20, 22).

Susceptibility is, therefore, not a binary state of believer vs. non-believer, nor a single unitary construct. It is better understood as operating across three analytically distinct yet interacting levels—dispositional, situational, and process—each capturing a different dimension of how individuals encounter, interpret, and act on misleading health information. At the dispositional level, susceptibility is a stable trait. Individuals differ in their baseline capacity to distinguish accurate from misleading information as a function of cognitive reflection, analytical reasoning, and literacy (14, 20, 23). At the situational level, it is a state; hence, even individuals with strong baseline discernment may be rendered vulnerable by message framing, emotional salience, source cues, prior exposure, or social endorsement (24, 25). At the process level, susceptibility unfolds sequentially across four stages: source selection, information selection, credibility evaluation, and reaction (19). Transitions between stages are neither automatic nor uniform. Li and Yang (18), in a meta-analysis of 28 RCTs, find that exposure produces only a small average effect on misbelief (*d* = 0.28). Because different factors govern each stage, improved detection does not necessarily prevent downstream diffusion at the reaction stage (17, 22, 26). These levels explain why individuals who can identify false information may still accept or share it and why single-factor accounts of susceptibility are inadequate.

#### 3.1.2 Measurement approaches

Research on susceptibility to health misinformation employs a variety of measurement approaches, reflecting the construct's multidimensional nature. Broadly, susceptibility has been operationalized as the endorsement of false beliefs, the capacity for accurate discernment, downstream behavioral responses, or as an inferred latent propensity based on response patterns. Each approach captures a different facet of the dispositional, situational, and process-level framework introduced in the preceding section.

*Belief and accuracy judgment measures*. The most common approach asks participants to rate the perceived accuracy of specific false claims (e.g., COVID-19 myths, vaccine misconceptions, folk beliefs about cancer) on Likert-type scales (4, 27–32). These measures capture static belief states but can conflate susceptibility with prior exposure or entrenched attitudes rather than evaluative capacity (14).

*Discernment-based measures*. Discernment measures conceptualize susceptibility as the ability to differentiate true from false health information. Participants evaluate balanced claim sets, enabling the calculation of difference scores, classification accuracy, or proportion-correct indices (14, 22). This separates truth recognition from false-belief endorsement, reduces ceiling effects, and provides a performance-based assessment (23). Recent applications extend this to health contexts using validated headline- or claim-based batteries (12, 33).

*Signal Detection Theory-based measures*. Signal Detection Theory (SDT) disentangles discrimination ability (how well individuals distinguish true from false information) from response bias [the tendency to judge information as true or false regardless of veracity; (15, 34)]. A specific form of response bias is myside bias, which refers to the tendency to evaluate information in ways that favor one's preexisting beliefs or group identity, thereby distorting accuracy judgments even when discrimination ability is intact. Discrimination ability is positively associated with cognitive reflection and open-minded thinking, and negatively associated with bullshit receptivity and conspiracy mentality, whereas myside bias is more closely linked to ideological congruence and partisan identity (15, 35).

*Attitudinal, intentional, and behavioral outcome measures*. Susceptibility has also been measured by downstream shifts in attitudes, intentions, or behaviors following exposure, such as sharing misinformation or fact-checking (22, 36). Such outcomes capture the functional consequences of exposure (18, 37). These measures capture real-world impact but often involve emotion, norms, and trust as confounders, hence complicating causal attribution.

*Latent profile and clustering approaches*. Latent profile and clustering methods identify heterogeneous susceptibility patterns, treating susceptibility as an inferred propensity rather than a uniform characteristic. Agley and Xiao (38) used latent profile analysis to identify distinct COVID-19 narrative belief profiles and found that lower trust in science was the strongest predictor of membership in misinformation-endorsing profiles. Álvarez-Gálvez et al. (27) further combine factor analysis with k-means clustering to segment individuals into profile groups, such as “convinced,” “hesitant,” and “skeptical,” regarding COVID-19 misinformation. Others use computational models to infer continuous latent susceptibility scores from large-scale sharing behavior and user characteristics (13). Complementing these approaches, Krishna (39) proposed a typology of four misinformation-susceptible publics—immune, vulnerable, receptive, and amplifying—which has since been empirically validated and applied to vaccine misinformation contexts (39). Amplifying publics, who both misperceive and spread misinformation, represent the most consequential profile.

### 3.2 Determinants of susceptibility to health misinformation

**Figure 1:**
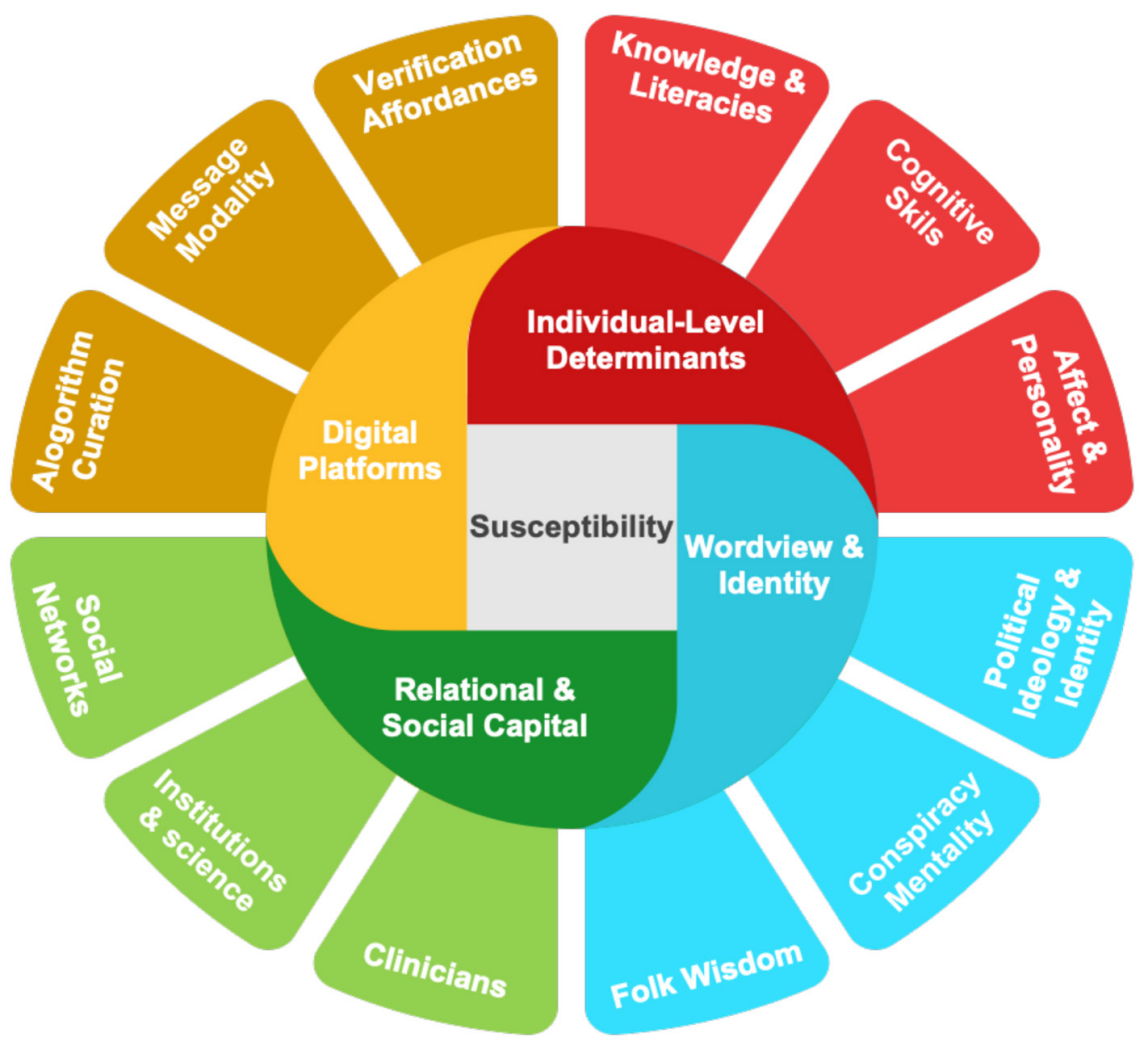
Multi-level determinants shaping susceptibility to digital health misinformation. Arrows indicate cross-level interactions among individual-level, relational, and platform/contextual factors that jointly determine susceptibility outcomes.

Susceptibility emerges from the interaction of individual, relational, and platform-level factors that collectively shape how people evaluate, accept, or resist misleading health information (see Figure 1).

#### 3.2.1 Individual-level determinants: literacies, cognition, personality, and identity

##### 3.2.1.1 Literacies and knowledge

Several distinct yet related literacy constructs emerge across the health misinformation literature as individual-level protective factors. These include health literacy (the ability to access, understand, appraise, and apply health information), eHealth literacy (the capacities exercised in digital and online environments), digital literacy (functional competence with digital tools and platforms), news literacy (the ability to evaluate news credibility and provenance), and media literacy (the broader ability to analyze media messages and production critically). Although these constructs differ in scope and measurement, they share a common protective mechanism. They support the ability to evaluate health claims accurately, which is one of the two main psychological pathways influencing susceptibility to health misinformation, along with the motivation for accurate reasoning (17).

Lower health literacy is associated with greater belief in false health claims and poorer detection of myths across diverse topics and populations (10, 14, 30, 33, 40–42). Further, reviews of infodemics and digital health more broadly identify low digital and eHealth literacy as key vulnerability factors (43, 44). Notably, Scherer et al. (14) find that susceptibility to health misinformation is cross-topically consistent, such that individuals susceptible to one health topic tend to be susceptible across others. However, the protective effect is not uniform across all types of false belief. Cheng and Nishikawa (40), in a survey of 1,488 Japanese adults, found that health literacy reduced belief in COVID-19 and vaccination misinformation but had no direct effect on conspiracy beliefs. These findings indicate that while low literacy is a reliable risk factor, high literacy is not a sufficient preventive measure, particularly when susceptibility is driven by motivated reasoning rather than limited evaluative capacity.

Self-perceived eHealth literacy further complicates this picture (39, 41, 45). Krishna et al. (39) report that self-reported eHealth literacy is not associated with reduced misperceptions following exposure to health misinformation. Similarly, Xie and Zhang (45) report that higher self-perceived eHealth literacy is associated with poorer accuracy in distinguishing AI-generated from human-written health misinformation. Overall, these results suggest that self-reported eHealth literacy reflects confidence more than actual skill (i.e., a Dunning–Kruger effect), which may lead to overconfidence and greater vulnerability in AI-driven information environments. Sirlin et al. (23) find that digital literacy predicts accuracy in detecting misinformation but shows weaker, inconsistent associations with restraint from sharing. Chan (46) similarly finds that news literacy improves discrimination and encourages authentication behaviors but does not consistently reduce sharing.

Hu et al. (47) identify a further paradox in media literacy's relationship to health misinformation. In a cross-sectional survey of 693 Chinese adults, higher media literacy was associated with better credibility discernment of health information. However, participants with higher media literacy scores exhibited a stronger continued influence effect of misinformation (CIEM)—that is, they continued to rely on prior misinformation in their judgments even after receiving a valid correction. Media literacy is thus a double-edged resource. It improves initial detection but may entrench resistance to correction precisely in those who are most skilled at identifying false information.

Overall, validated health and digital literacy help people evaluate information and reduce susceptibility to misinformation, but they have limits. They do not consistently protect against conspiracy beliefs driven by motivation, nor do they reliably restrain sharing behavior. Moreover, self-perceived literacy may lead to overconfidence and more vulnerability. These findings indicate that literacy interventions should also address motivation, social context, and metacognitive awareness for lasting impact.

##### 3.2.1.2 Cognitive abilities and styles

Research shows that analytic thinking, including cognitive reflection, numeracy, and open-mindedness, improves accuracy in discerning truth and reduces susceptibility to health misinformation (11, 14, 15, 22, 28, 35, 42). Individuals with higher cognitive reflection and conscientiousness are more likely to agree with expert assessments of COVID-19 content (48). Moreover, a higher need for cognition buffers against the misperception-forming effects of low-effort news engagement, such as “news-finds-me” perceptions (i.e., the belief that one can stay informed without actively seeking news), on the formation of misperceptions (11, 49, 50).

However, the protective role of analytic thinking is conditional on the motivational context in which it operates. Evidence consistently shows that myside bias (i.e., the tendency to evaluate information in ways that confirm pre-existing beliefs) predicts susceptibility more strongly than numeracy or cognitive reflection (51, 52). When analytic capacity is deployed in the service of identity-confirmation rather than evidence evaluation, higher cognitive ability can reinforce rather than reduce susceptibility.

In addition, the interaction between motivation and capacity varies with processing style and media use. Individuals with a high Need for Cognition (NFC) and Faith in Intuition (FI) are more vulnerable to misinformation on social media and in alternative health sources, as their analytical thinking is directed toward supporting identity-based falsehoods (8). Conversely, those with low faith in intuition may also form misperceptions through analytical processing of high-volume, misleading content (53). At the level of processing mode, heuristic processing amplifies the link between exposure to misinformation and acceptance. In contrast, systematic processing provides no consistent protection, thus confirming that processing mode alone cannot compensate for motivational orientation (54).

These dispositional dynamics are further influenced by situational variables. In contexts where issue salience is high, intensive information seeking often leads to information overload, which subsequently contributes to the development of misperceptions and intentions to share them. The NFC partially moderates the link between information seeking and overload, while FI affects the progression from misperception formation to sharing intentions (49, 53).

##### 3.2.1.3 Personality, affect, and emotional mechanisms

Evidence on personality traits is comparatively limited but generally indicates that dispositional differences modestly shape both susceptibility to and sharing of health misinformation. Studies of social media engagement show that conscientiousness, openness, and empathic concern are associated with lower engagement, particularly “liking” of health misinformation. In contrast, grandiose narcissism and ideologically motivated reasoning tendencies are more predictive of active propagation behaviors (55). Beyond personality alone, Rivers et al. (56) demonstrate that, among Japanese youth, personality traits, combined with facets of national identity, explain meaningful variance in both vaccine hesitancy and susceptibility to COVID-19 misinformation, even after accounting for gender, political ideology, and trust in government pandemic responses.

In contrast, affective and emotional processes play a more central role. Experimental evidence shows that anxiety mediates the effects of multimodal disinformation, particularly video-plus-text formats, on vaccine-related misperceptions (57). Related work shows that emotionally vivid formats, such as health-related deepfakes, increase misperceptions and suppress fact-checking (12). Health-related anxiety, worry, and preexisting misbeliefs are also strong predictors of misinformation acceptance (29, 58). In a two-wave panel study, Jiang et al. (59) show that worry and heightened risk perception lead to negative COVID-19 information-seeking experiences, which in turn reduce fact-checking. This indirect effect is strongest among those with a greater propensity to trust COVID-19 misinformation.

##### 3.2.1.4 Worldview, ideology, identity, and epistemic beliefs

Worldview, ideology, identity, and epistemic beliefs are important determinants of susceptibility (2, 15, 17, 35, 56). In the United States and other high-income contexts, studies report that conservative or right-leaning political orientations, or heavier reliance on conservative news sources, are often associated with greater endorsement of politicized COVID-19 and vaccine misinformation (4, 11, 15, 22, 27, 31, 40, 56, 60–62). Additionally, conspiracy mentality and bullshit receptivity are robustly associated with lower truth sensitivity and higher misbelief (15, 31, 35).

Favorable attitudes toward alternative medicine predict beliefs about cross-topic health misinformation (14). Medical folk wisdom mediates the relationship between exposure to alternative health media and beliefs about vaccine misinformation, with institutional trust operating as a moderator (32). Among unvaccinated Black Americans, conservative ideology, conspiracy mindset, religiosity, and racial consciousness in healthcare are associated with stronger COVID-19 vaccine misinformation endorsement and lower vaccine acceptance (63).

At the media interface, the “news-finds-me” (NFM) perception predicts higher COVID-19 misperceptions. It also mediates the link between social media use and misinformation, with the Need for Cognition (NFC) attenuating this pathway (50, 64). Research also suggests that tabloid/alternative media environments, along with uncertainty about accuracy, can amplify agreement with sensationalist COVID-19 disinformation (22, 65, 66).

#### 3.2.2 Relational and social-capital factors

Relational and social capital factors, particularly trust in scientists, clinicians, governmental bodies, the media, and peers, are among the most robust predictors of susceptibility, often outweighing the content of the information itself (17, 22, 38). This section examines trust as defined by four key relational areas: (1) trust in science, medicine, and healthcare professionals; (2) trust in government and public authorities; (3) trust in media and interpersonal sources; and (4) support from social and community networks.

A conceptual clarification is warranted here. While trust perceptions occur at the individual level, the factors in this section are classified as relational because they depend on social relationships (e.g., patients–clinicians, citizens–institutions), rather than on personal beliefs or worldviews such as conspiracy mentality or alternative medicine. Trust is inherently directed toward an external source, and its impact on susceptibility depends on the perceived credibility of that source.

##### 3.2.2.1 Trust in science, medicine, and doctors

Lower trust in scientific and medical authorities is one of the strongest correlates of susceptibility (17, 22, 38, 55, 62, 67). In a U.S. national survey on statins, cancer, and HPV misinformation, lower institutional trust predicted endorsement of false claims across all topics (14). In COVID-19 contexts, higher trust in scientists and medical experts was associated with lower belief in misinformation and stronger vaccination intentions (22, 62, 63). Trust in medical science also predicts behavior. In a Twitter-simulation study, individuals with greater trust in medical science were less likely to “like” health misinformation (55). Trust thus constrains both belief formation and propagation.

These findings align with models that position trust in science and medicine as central to susceptibility (2, 17). As a mediator, trust links identity and ideology to misbelief. As a moderator, it determines whether exposure to the same message leads to acceptance or rejection. Among all sources, trust in clinicians emerges as a particularly robust protective factor. Using a large, nationally representative U.S. sample, Horoszko et al. (67) show that trust in doctors is strongly and consistently associated with lower endorsement of COVID-19 misinformation, even after adjusting for sociodemographic characteristics.

##### 3.2.2.2 Trust in government and public authorities

Across diverse national contexts, higher trust in government and public health authorities is generally associated with lower susceptibility to health misinformation. Nationally representative surveys show that greater trust in governmental institutions predicts lower endorsement of COVID-19 myths in Lebanon (36), Norway (61), and Spain (27). Evidence from the United States similarly indicates that belief in COVID-19 vaccine misinformation is systematically linked to lower trust in government and public institutions (62).

However, the protective role of government trust is highly context-dependent and politically contingent. In highly politicized information environments such as the United States during the COVID-19 pandemic, trust in government does not operate uniformly. Reliance on partisan media networks, especially conservative outlets, has been associated with greater endorsement of misinformation, even in the presence of official governmental messaging (60). Evidence further indicates that levels of trust vary across government entities and that partisan affiliations influence whether trust serves as a protective factor or contributes to polarization (62).

##### 3.2.2.3 Trust in media and interpersonal sources

Trust in media and interpersonal sources also shapes susceptibility. Trust in social media, interpersonal communication, and informal authorities is consistently associated with greater vulnerability (36, 58, 61). In the United States, consuming conservative pundit-led news correlates with greater acceptance of misinformation, whereas certain social media news sources show neutral or negative associations with misbelief (60). Research conducted in Japan demonstrates that reliance on social media for COVID-19 information correlates with lower health literacy and greater susceptibility to misinformation and conspiracy theories (40).

In contrast, reliance on legacy and professional news sources generally exhibits a protective role. These findings are observed in Lebanon, Norway, and Japan, where higher consumption of traditional news is associated with lower endorsement of COVID-19 myths (36, 40, 61). These findings highlight that media trust is not monolithic. Trust in professionally curated news and expert sources tends to function as a protective factor. In contrast, trust in loosely regulated, peer-driven, or identity-aligned channels often increases susceptibility to health misinformation.

##### 3.2.2.4 Social support and community networks

Social support and community ties are central components of social capital, yet their relationship to health misinformation is inherently ambivalent. Evidence from a large U.S. survey shows that trust in healthcare clinicians consistently protects against COVID-19 misinformation across racial and ethnic groups, whereas interpersonal and community-based social support exhibits heterogeneous effects (67). Social support reduces susceptibility when networks value accurate information but amplifies it when networks circulate or legitimize misinformation.

Furthermore, studies of misinformation diffusion highlight how social ties amplify sharing behavior, often independent of belief accuracy. A review of COVID-19 misinformation sharing shows that stronger tie strength, altruistic motives, and sociability are frequently associated with increased sharing, driven by intentions to help, warn, or maintain relationships rather than to deceive (68). These findings suggest that social capital functions as a double-edged resource. It can buffer individuals against misinformation when aligned with credible expertise, but magnify harm when relational trust and prosocial motives operate within misinformed networks.

#### 3.2.3 Platforms, environment, and message-level features

Beyond general media choice, platform architectures, algorithmic environments, and message affordances all play distinct roles in shaping susceptibility to health misinformation. A growing body of research highlights the influence of passive and algorithmically curated news environments, often operationalized through the “news-finds-me” (NFM) perception. U.S. research shows social media use indirectly predicts COVID-19 misperceptions via NFM perceptions rather than through use alone (64). Related research demonstrates that NFM operates partly through information avoidance, with the need for cognition attenuating this indirect pathway (50).

Information overload is a key platform mechanism. Intensive health information seeking increases overload, which predicts misperceptions, with Need for Cognition (NFC) moderating the seeking-to-overload link (49). Misperceptions mediate the link between social media seeking and sharing intention, with Faith in Intuition (FI) acting as a moderator (49, 53). Latent models of social media use reveal pronounced clustering of vulnerabilities across political, professional, and geographic groups (13).

Platform design cues and message modality also shape susceptibility independently of content accuracy. Experimental evidence shows that verification affordances, such as checkmarks or flags, exert stronger effects on perceived credibility and behavioral intentions than author credentials, and that these effects are moderated by users' social media self-efficacy (37). Video-based misinformation elicits stronger vaccine misperceptions than text-only formats through heightened emotional engagement and perceived relevance (57). Health-related deepfakes substantially increase misperceptions and suppress fact-checking, especially among those with high issue salience and illusory accuracy motivation (12). AI-generated health messages are generally perceived as more persuasive than human-written ones (45). In AI-driven recommendation systems, perceptions of transparency, fairness, and accountability further shape whether users process health information heuristically or systematically, influencing credibility judgments (69).

### 3.3 Cross-level interaction effects

This review confirms that susceptibility to health misinformation results from complex interactions across multiple levels, including individual cognition, message design, media environments, social identity, and trust orientations. Rather than acting in isolation, these factors shape vulnerability, depending on how they align within specific contexts (see Table 1). Below are the most prevalent interaction effects.

**Table 1:** Multi-level determinants and interaction patterns influencing health misinformation susceptibility.

Primary factor [Representative studies]	Typical direction (Susceptibility)	Interaction patterns	Key outcomes	Implications
Individual–Cognitive/Skill				
**Health literacy (objective)** (10, 14, 30, 31, 33, 40–42)	Protective (higher literacy → lower susceptibility)	Effects persist beyond demographics/media use; does not fully eliminate conspiracy belief (40)	Lower myth endorsement; Higher discernment	Prioritize performance-based literacy measures; treat as core protective resource, not a ‘silver bullet.'
**eHealth literacy (objective vs. self-perceived)** (39, 45)	Mixed: objective protective; self-perceived can mislead	Self-perceived competence may not reduce misperceptions; can backfire in AI-content contexts (45)	Misperceptions; sharing; failure to correct	Measure actual skill + calibration gaps, not self-report only.
**Media, digital & news literacy** (23, 46, 47)	Generally protective for discernment; weaker for sharing restraint	Accuracy discernment ≠ sharing restraint (evaluation–behavior gap); higher media literacy can amplify continued influence of misinformation after correction — the CIEM paradox (47)	Better verification/source checking; variable sharing restraint	Separate discernment from propagation outcomes; include behavioral measures; address CIEM with metacognitive calibration.
**Topic-specific knowledge (objective)** (30, 33)	Protective	Works when measured objectively; may interact with overconfidence	Lower belief in domain myths	Include objective knowledge measures in domain-specific interventions (e.g., nutrition, cancer).
**Overconfidence/miscalibration (‘pseudo-expert' pattern)** (41, 45)	Risk-enhancing	Dunning–Kruger: low objective skill + high confidence → worst detection	Poor identification; resistance to correction; higher sharing	Add metacognitive calibration indices; target ‘pseudo-experts' with humility- and feedback-based strategies.
**Cognitive reflection/analytic thinking/AOT** (11, 14, 15, 20, 22, 28, 35, 42, 52)	Protective (on average)	Improves truth sensitivity more than ‘global skepticism'; myside bias predicts susceptibility more strongly than numeracy or cognitive reflection (51, 52); high cognitive ability can reinforce misbelief when deployed in service of identity-confirmation	Better veracity discernment; lower misbelief	Use SDT/discernment designs to distinguish discrimination ability from response bias; address myside bias directly.
**Need for Cognition (NFC)** (8, 11, 49, 50)	Often protective, but can backfire	Buffers NFM orientations; may increase susceptibility when paired with poor-quality content + Faith in Intuition (FI)	Discernment; misperceptions; sharing via overload pathways	Treat NFC as context-dependent; model NFC × information quality and NFC × FI.
**Faith in Intuition (FI)** (8, 49, 53)	Mixed/context-dependent	Moderates social media seeking → misperceptions; amplifies overload-to-misperception chains; effects can be counterintuitive	Misperceptions; sharing intentions	Test conditional effects and interaction with exposure/content quality; avoid treating FI as uniformly harmful.
**Heuristic vs. systematic processing** (54)	Heuristic risk-enhancing; systematic not reliably protective	Heuristic processing strengthens exposure → acceptance; systematic processing may not offset motivated/identity-driven processing	Acceptance of misinformation following exposure	Reduce reliance on superficial cues; strengthen epistemic vigilance, not just ‘more thinking.'
**Situational motivation/involvement** (39)	Often risk-enhancing post-exposure	High motivation can increase both misperceptions and sharing when the information environment is polluted	Misperceptions; amplification	Motivation is not always protective; context + quality determine whether involvement helps or harms.
Individual–Affective/Personality				
**Personality traits (conscientiousness, openness, empathy; narcissism)** (55, 56)	Modest, mixed (traits shape engagement/propagation)	Traits relate more to behavioral propagation than belief accuracy; narcissism and motivated reasoning predict active propagation	Liking/sharing; propagation patterns	Use personality for understanding propagation propensity and audience segmentation rather than belief correction.
**Anxiety/worry/risk perception** (29, 57–59)	Generally risk-enhancing (especially under vivid formats)	Emotions mediate modality effects (57); worry degrades fact-checking via negative seeking experiences (59); social media use → worry → misbelief pathway (58); faith in scientists moderates this pathway	Misperceptions; reduced fact-checking	Model both trait anxiety and state emotion; design interventions that regulate affect and support verification.
15.6-7.4,-39499pt**Deepfakes/emotionally vivid misinformation** (12)	Risk-enhancing	Effects strongest when issue relevance is high and “accuracy motivation” is illusory	Higher misperceptions; lower fact-checking	Treat AI-manipulated modalities as high-risk; require tailored detection and platform safeguards.
Individual–Worldview/Identity				
**Political ideology (conservative/right-leaning)** (4, 11, 15, 22, 27, 31, 40, 56, 60–62)	Often risk-enhancing for politicized health misinformation (context-dependent)	Effects persist beyond cognitive style in some settings; shaped by media ecology; conservative media reliance amplifies misbelief even in presence of official messaging (60); conservative ideology, conspiracy mindset, religiosity, and racial consciousness in healthcare jointly predict stronger vaccine misinformation endorsement among unvaccinated Black Americans (63)	Misbelief; lower detection; vaccine outcomes	Treat ideology as a contextual moderator; emphasize trusted messengers and depolarized framing.
**Conspiracy mentality/bullshit receptivity** (15, 31, 35)	Strong risk-enhancing	Linked to lower truth sensitivity and higher misbelief across health domains; interacts with ideological and partisan identity to reduce discrimination ability (15, 35)	Misbelief; lower discrimination ability	Include conspiracy-related constructs; interventions may need identity-safe and trust-repair components.
**Alternative medicine beliefs & medical folk wisdom** (14, 32, 66)	Risk-enhancing	Folk wisdom mediates alternative health media → misbelief pathway; institutional trust conditions the pathway (32)	Vaccine misinformation belief	Measure folk wisdom explicitly; address underlying health belief systems, not only factual corrections.
**National/cultural identity** (56)	Context-dependent	Combines with personality/ideology to structure susceptibility and vaccine hesitancy	Susceptibility; vaccine hesitancy	Build culturally grounded interventions; segment by identity-relevant concerns and values.
15.6-7.4,-39499pt**News-Finds-Me (NFM) worldview** (50, 64)	Risk-enhancing	Mediates social media use → misperceptions via exposure/avoidance; NFC attenuates effects (50); linked to information avoidance	Misperceptions; avoidance; passive reliance	Treat NFM as an epistemic stance to target; promote active seeking and verification routines.
Relational–Trust/Social capital				
**Trust in science/medicine** (2, 14, 17, 22, 38, 55, 62, 63, 67)	Protective	Operates as mediator/moderator in integrative models; constrains both belief formation and propagation (55)	Lower misbelief; better discernment; less liking/sharing	Treat as social–epistemic capital; prioritize trust-building and credible messenger strategies.
**Trust in clinicians (doctors)** (67)	Strongly protective	Robust across racial/ethnic subgroups; among the strongest and most consistent individual protective factors in nationally representative data	Lower endorsement of misinformation	Leverage clinician communication and patient–doctor relationships as primary intervention channels.
**Trust in government/public authorities** (27, 36, 56, 60–62)	Protective on average; politically contingent	Effects vary by polarization and agency-level trust; partisan affiliations influence whether trust protects or contributes to polarization (62)	Misbelief; vaccine misinformation endorsement	Disaggregate ‘government trust' by agency level and political context; avoid assuming uniform protection.
**Trust in media & interpersonal sources** (33, 36, 40, 60, 61, 66)	Differentiated: legacy/professional protective; social/peer/clerical often risk	Effects depend on platform and stream quality; can cluster with low literacy; trust in social media consistently associated with greater vulnerability	Myth endorsement; verification behavior	Measure where trust is placed (legacy vs. social vs. alternative vs. clerics), not ‘media trust' globally.
15.6-7.4,-51499pt**Social support/community networks** (67, 68)	Mixed (“double-edged”)	Protective when network norms are evidence-aligned; risk when networks legitimize misinformation; tie strength and prosocial motives amplify sharing independent of accuracy (68)	Misbelief; sharing diffusion	Measure support source + network norms; include interventions targeting peer norms and prosocial sharing motives.
Contextual–Platform/Algorithm				
**Algorithmic/digital environments** (13, 49, 53)	Generally risk-enhancing	Seeking → overload → misperceptions → sharing; moderated by NFC/FI; passive exposure via algorithmically curated feeds increases reliance on social endorsement cues	Misperceptions; sharing intentions	Model overload and passive feeds as susceptibility pathways, not just ‘time on social media.'
**Platform cues (verification flags) & social media self-efficacy** (37)	Cue-dependent (can reduce or increase perceived credibility)	Verification cues shape credibility and sharing intentions; effect moderated by social media self-efficacy; stronger than author credentials alone	Credibility; sharing; compliance intentions	Address cue interpretation and confidence-driven heuristic reliance in design and literacy interventions.
**AI systems (recommendation & content generation)** (45, 69)	Context-dependent	Perceived fairness, accountability, and transparency (FAccT) shapes heuristic vs. systematic processing; AI-generated content perceived as more persuasive than human-written (45)	Credibility; diagnostic accuracy	Require transparency and user education to prevent over-reliance; treat AI trust as a processing determinant.

AOT, Actively Open-minded Thinking; CIEM, Continued Influence Effect of Misinformation; FAccT, Fairness, Accountability, and Transparency; FI, Faith in Intuition; NFC, Need for Cognition; NFM, News-Finds-Me; SDT, Signal Detection Theory.

1. Cognitive styles interact with media exposure and information quality. High need for cognition (NFC), typically protective, can backfire when paired with strong FI and poor-quality content, deepening misinformation processing (49, 53).

2. Epistemic worldviews shape platform engagement: the “news-finds-me” (NFM) belief interacts with algorithmic feeds, hence increasing exposure to misinformation. NFM mediates social media use and COVID-19 misperceptions, attenuated by higher NFC (50, 64).

3. At the message level, video and deepfake misinformation increase misbelief through anxiety and perceived relevance, amplified among those with high issue involvement—indicating that motivated reasoning and emotional vividness jointly shape susceptibility (12, 57).

4. Trust operates conditionally: exposure to alternative health content predicts vaccine misinformation via medical folk wisdom, moderated by institutional trust (32). Negative information-seeking experiences reduce fact-checking, especially among those predisposed to trust misinformation (59).

5. Social identity and structural factors further shape vulnerability. Among unvaccinated Black Americans, factors such as conservative ideology, religiosity, conspiracy mindset, and racial consciousness interact with experiences of medical mistrust to predict stronger belief in vaccine misinformation and lower vaccine confidence (63, 67).

6. Finally, platform features and AI-mediated environments introduce additional layers of interaction. Verification tags influence perceived credibility and sharing intentions, but their impact is moderated by users' digital self-efficacy (37). Higher self-perceived eHealth literacy can increase susceptibility in AI settings through overconfidence in evaluation (45).

## 4 Discussion

### 4.1 What makes health misinformation distinct

Health misinformation differs structurally from political misinformation in ways with direct implications for how susceptibility should be theorized and measured. A useful organizing lens is uncertainty management theory (70), which holds that individuals respond to health-related uncertainty through a range of cognitive, affective, and behavioral strategies (i.e., seeking, avoiding, or reframing information depending on how threatening or manageable the uncertainty feels). Health misinformation exploits this uncertainty by providing false certainty and emotional comfort when people feel most vulnerable.

Three structural features distinguish health misinformation from its political counterpart. First, health claims are bound up with fear, embodied risk, and personal stakes, including mortality, bodily integrity, and responsibility for dependents. These features make health claims inherently prone to anxiety-driven processing. Emotional arousal heightens vigilance but simultaneously narrows attention, increases reliance on heuristics, and reduces engagement in corrective behaviors such as fact-checking.

Second, health misinformation is distinctive in the central epistemic role that clinicians and medical professionals play. Trust in doctors functions as a potent and well-documented buffer against misbelief. Unlike diffuse trust in science or government, clinician trust is interpersonal and relational, grounded in lived experience. This makes health misinformation uniquely sensitive to disruptions in clinical relationships and helps explain why rebuilding interpersonal trust in healthcare settings can produce downstream effects on beliefs and behavior that impersonal corrections cannot replicate.

Third, health misinformation is shaped by alternative medicine beliefs and folk epistemologies that have no direct equivalent in political misinformation. Beliefs in natural remedies, traditional practices, or biomedical skepticism provide coherent, identity-affirming frameworks that compete with institutional health guidance. Corrective information may be evaluated not only on its evidential merits but also against a competing epistemological framework that the individual regards as equally or more legitimate.

### 4.2 Susceptibility is configurational, not additive

A central insight emerging from this synthesis is that susceptibility to health misinformation is configurational rather than additive. Single-factor explanations (e.g., low literacy, weak analytic skills, or high social media use) often fail to account for the complex ways in which individual dispositions, relational trust, and media environments combine to produce vulnerability. High cognitive engagement, for example, does not uniformly reduce misbelief. When analytic motivation is coupled with strong Faith in Intuition (FI), identity-congruent priors, or sustained exposure to low-quality information environments, cognitive resources may be deployed to elaborate rather than correct misinformation. What appears as “high cognition” in isolation thus becomes a liability when aligned with intuitive trust and permissive media ecologies.

This perspective recasts susceptibility as an issue of how different factors dynamically align. Analytic capacity protects when applied within environments that reward accuracy and verification, but it can magnify error when embedded in networks or platforms that normalize misinformation. Understanding susceptibility, therefore, requires attention to how cognitive resources are mobilized, not merely whether they are present.

### 4.3 Trust as social–epistemic capital

Trust acts as social–epistemic capital that often surpasses literacy or cognitive ability, shaping which sources are deemed credible, what claims are believed, and which corrections are considered. In health misinformation, three distinct types of trust exist: interpersonal (trust in clinicians and providers), institutional (trust in medical science and authorities), and network-based (trust in peers and communities). Interpersonal trust offers strong protection against misinformation because it anchors judgment and resists identity-based challenges. Institutional trust is more vulnerable to politicization and historical grievances. Network-based trust can increase risk, as misinformation shared within trusted groups is amplified. The key variable is trust alignment—whether trust is placed in sources that reliably guide toward accurate information.

### 4.4 Platforms as susceptibility multipliers

Digital platforms do not merely host misinformation. Rather, they multiply susceptibility by shaping how information is encountered, processed, and acted upon. Algorithmic passivity can be captured by orientations such as the belief that news will “find” the user, thereby reducing active verification and increasing reliance on socially endorsed cues. AI-enabled realism further intensifies these dynamics. Highly polished, fluent, or audio visually rich content increases perceived credibility. When combined with overconfidence in one's digital or health literacy, these affordances can undermine detection accuracy and suppress corrective behaviors. Importantly, platform affordances often override individual skills. Even highly motivated or knowledgeable users can be misled when cues of realism, endorsement, or legitimacy are sufficiently strong.

### 4.5 Implications for stakeholders and affected communities

The synthesis clarifies that effective responses to health misinformation require targeted strategies for different stakeholders.

- Patients and the public: Vulnerability is shaped by social and emotional factors, not just knowledge gaps. Communication must avoid stigma, address emotions, and be culturally sensitive, especially for those with lower literacy or trust.
- Clinicians: Clinical relationships are a key defense. Building trust through patient-centered, culturally responsive care and adequate consultation is vital for countering misinformation.
- Public health and policymakers: Simply providing more information is insufficient. Consistent, transparent messaging and policies that reinforce institutional trust are needed to reduce susceptibility.
- Platform designers: Modifying algorithms to slow sharing, reduce overload, clarify uncertainty, and enhance verification can lessen misinformation risks. Overreliance on verification cues should be avoided to prevent false confidence.
- Marginalized communities: Addressing medical mistrust requires not just accurate information, but also systemic accountability and genuine community engagement to reduce the appeal of alternative networks.

### 4.6 Implications for intervention design

Effective interventions must be tailored to the specific factors and cognitive processes that drive susceptibility to misinformation. Rather than applying generic solutions, strategies should directly address the underlying determinants—whether they are low analytical engagement, identity-driven reasoning, lack of trust, platform design, or social network influences. For example, calibration and accuracy prompts are best for correcting overconfidence and inattentiveness; value-affirming framing and trusted community messengers are more effective when identity or belief systems are involved; rebuilding trust requires transparent, clinician-centered communication; platform-driven vulnerabilities demand design changes such as slowing sharing and labeling uncertainty; and network-driven susceptibility is best countered through norm correction and peer modeling (see Table 1).

### 4.7 Implications for measurement and theory

This review highlights the need to reconceptualize susceptibility. Belief alone is insufficient as an outcome because susceptibility unfolds across stages, including exposure, interpretation, confidence, sharing, and behavioral response. Measuring only belief obscures important mechanisms and intervention leverage points. In addition, future research should adopt multi-stage outcome models, incorporate interaction terms by design, and move beyond single-factor predictors. Theoretical frameworks must recast vulnerability as emerging from the alignment of individual dispositions, trust relations, and media environments. Longitudinal designs and behavioral measures are especially needed to capture how susceptibility evolves over time and translates into real-world health decisions.

### 4.8 Limitations of the evidence base

The current synthesis is limited by its exploratory, integrative (rather than systematic) nature, reliance on cross-sectional studies, lack of qualitative research, and focus on self-reported attitudes rather than real-world behavior. Furthermore, its Western and COVID-19-centric orientation restricts broader applicability. Additionally, crisis conditions like the pandemic intensify anxiety, heuristic processing, and trust dynamics, fundamentally altering information environments and the effectiveness of platform moderation. While some mechanisms—such as cognitive reflection and conspiracy beliefs—remain stable across contexts, others—including emotional arousal and erosion of institutional trust—are heightened during crises. To advance understanding, future research should contrast susceptibility across crisis and non-crisis settings to clarify which predictors are context-dependent versus universally stable.

## 5 Conclusion

This review presents a multi-level synthesis that informs both theoretical approaches and the design of effective interventions. Effective intervention will require approaches that extend beyond fact-checking to strengthen trust, calibrate confidence, promote reflective processing, support clinicians and credible messengers, and redesign digital environments to reduce frictionless exposure and virality. Future research should employ longitudinal, mixed-methods, and cross-level designs to trace how susceptibility evolves and how interventions can best disrupt the pathways linking exposure to real-world health behavior.
